# Natural Pigments Production and Their Application in Food, Health and Other Industries

**DOI:** 10.3390/nu15081923

**Published:** 2023-04-16

**Authors:** Eleonora Di Salvo, Giovanna Lo Vecchio, Rita De Pasquale, Laura De Maria, Roberta Tardugno, Rossella Vadalà, Nicola Cicero

**Affiliations:** 1 Departement of Biomedical and Dental Sciences and Morphofunctional Imaging, University of Messina, 98168 Messina, Italy; edisalvo@unime.it (E.D.S.); rossella.vadala@unime.it (R.V.); 2 Department of Veterinary Sciences, University of Messina, 98168 Messina, Italy; 3 Department of Pharmacy-Drug Sciences, University of Bari, 70121 Bari, Italy; 4 Science4life srl, University of Messina, 98168 Messina, Italy

**Keywords:** pigments, food, bacteria, nutraceuticals, health

## Abstract

In addition to fulfilling their function of giving color, many natural pigments are known as interesting bioactive compounds with potential health benefits. These compounds have various applications. In recent times, in the food industry, there has been a spread of natural pigment application in many fields, such as pharmacology and toxicology, in the textile and printing industry and in the dairy and fish industry, with almost all major natural pigment classes being used in at least one sector of the food industry. In this scenario, the cost-effective benefits for the industry will be welcome, but they will be obscured by the benefits for people. Obtaining easily usable, non-toxic, eco-sustainable, cheap and biodegradable pigments represents the future in which researchers should invest.

## 1. Introduction

Natural pigments not only provide color but also offer potential health benefits as interesting bioactive compounds. These compounds have a variety of applications [1].

Studies have shown that synthetic dyes can have negative effects on both human health and the environment. Synthetic dyes are non-biodegradable, potentially carcinogenic, and can alter the taste of food. Many countries have banned the consumption of various dyes, such as Blue FCF, Blue No.1 and No.2, due to their toxicity.

**Figure 1:**
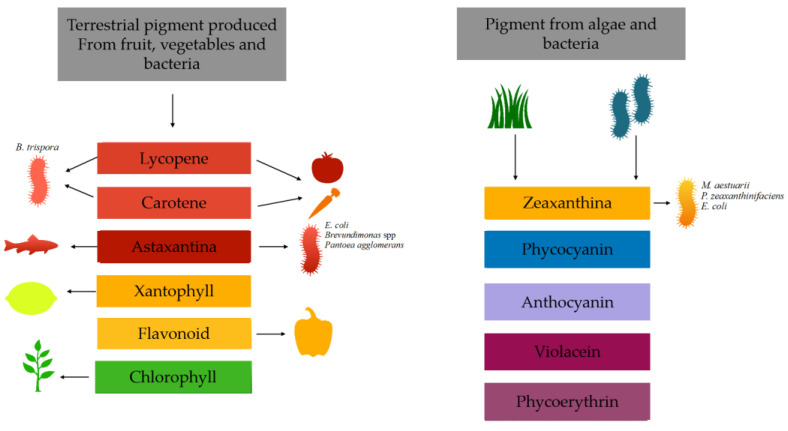
Examples of different natural pigments extracted from biomass and their sources.

There are two main sources of natural pigments: plants and microorganisms (Figure 1). However, using pigments from plants has several disadvantages, such as sensitivity to light, heat or pH and low water solubility. On the other hand, microbial pigments can be produced quickly and easily in a culture medium, which can even be waste. Unlike plant-based pigments, they are not affected by weather conditions. Therefore, the production of microbial pigments is an emerging research field with potential for various industrial applications [2]. This article emphasizes the importance of discovering natural microbial bioactive pigments from waste. Because there is a wide variety of pigments and natural sources with different properties and structures, there is no standardized method to obtain natural dyes [3].

## 2. Pigments Produced by Microorganisms from Waste

The demand for natural food additives has led to an increased interest in pigment production from microbial sources. This is due to the potential health hazards associated with synthetic dyes, some of which are teratogenic and carcinogenic [4,5]. Microbial pigments have proven to be an eco-friendly and non-toxic alternative, leading to their rapid replacement of synthetic dyes [6]. Microbial pigments have proven to be an eco-friendly and non-toxic alternative, leading to their rapid replacement of synthetic dyes [7]. Optimizing the growth conditions of microbes can further enhance pigment production. Factors such as the type of nitrogen and carbon source, carbon/nitrogen ratio, chemical supplements, aeration rate, agitation, light, and temperature and pH of the culture all play a role in microbial growth and pigment production [8,9]. Moreover, microbial pigments offer several medicinal benefits such as anticancer, antioxidant, antimicrobial, immunosuppressive, anti-inflammatory and antiproliferative activities. They are considered generally recognized as safe (GRAS) and are being scrutinized as a readily available source of coloring agents to replace synthetic chemical pigments. With genetic engineering, microbial pigment production can be greatly increased compared to the scaling-up methods of chemists. Microbes are more versatile and productive in the industrial-scale production of natural pigments and dyes. The fermentation process has been improved by genetic engineering, and further research on non-toxic microbial pigments can revolutionize the economics of microbial pigments [10]. Food wastes represent an important source of natural pigments both because they naturally contain pigments that can be extracted but also because they can be used as a fermentation substrate for the production of pigments by microorganisms. In this review techniques for the production of natural pigments using food waste and by-products of the agri-food industry as a fermentation substrate are reported, and their applications are discussed. As can be seen in Table 1, there are numerous microorganisms capable of producing natural pigments starting from different types of waste.

### 2.1. Carotenoids

Carotenoids are a group of fat-soluble pigments with a heterogeneous composition, varying in color from yellow to orange to red. They are made up of different isoprene units linked together to form a cyclic structure, and the modification of this structure determines the wide variety of compounds that can be formed [29]. Although carotenoids are commonly found in plant sources, they can also be produced by various microorganisms, including microalgae, cyanobacteria, fungi, bacteria and archaea. While traditional methods for producing carotenoids involve expensive synthetic processes or extraction from plant products, microbial carotenoids have become a promising source of natural carotenoids. This is because microorganisms can use low-cost agro-industrial wastes as substrates, reducing overall production costs. In addition, the fermentation process for producing microbial carotenoids is simple to manage and monitor, resulting in shorter processing times. These pigments have a strong antioxidant activity, which is determined by their unique chemical structure. Carotenoids are composed of a long chain of carbon atoms (made up of 35–40 atoms and called a polyene chain), often ending in a ring.

Carotenoid pigments are classified based on the structure of their chain into two classes:

- Xanthophylls, consisting of chains containing oxygen atoms; this group includes astaxanthin, lutein and zeaxanthin
- Carotenes are made up of oxygen-free molecules and are made up only of hydrogen and, in addition to carbon. The best-known classes are toluene, lycopene and carotene, which gives its name to the class [30].

**Figure 2:**
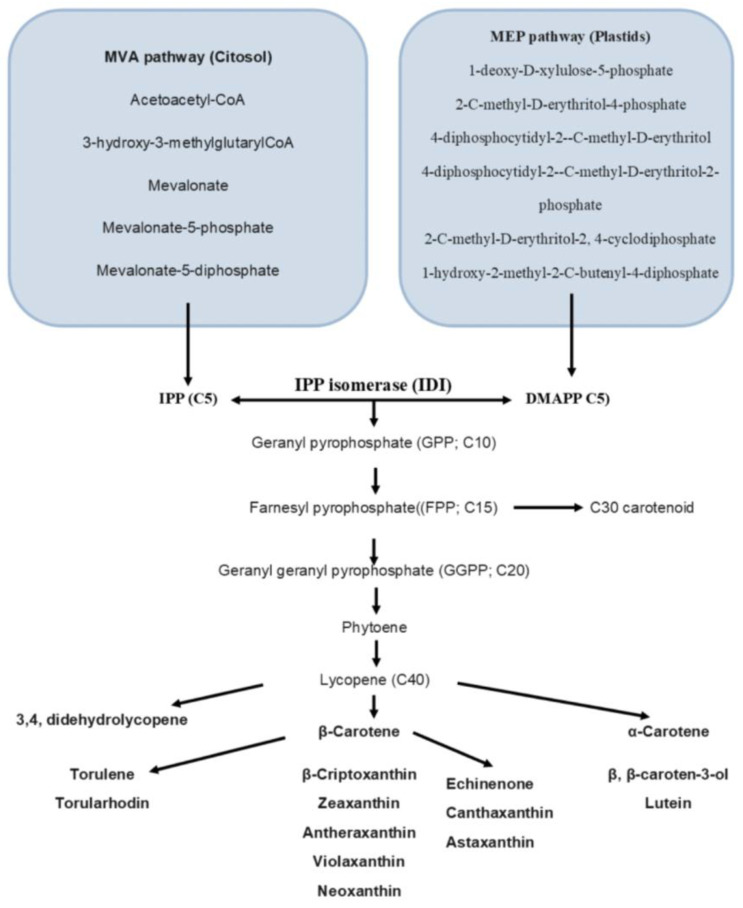
Schematic representation of selected pathways of carotenoid biosynthesis in microorganisms. The mevalonate (MVA) and 2-C-methyl-d-erythritol 4-phosphate (MEP) pathways.

Carotenoids belong to the group of terpenes, which includes countless classes of metabolites, both primary and secondary. Despite the structural and functional diversity, all isoprenoids originate from a common 5-carbon precursor, isopentenyl-5-pyrophosphate (IPP), enzymatically interconvertible into its isomer dimethylallyl–pyrophosphate (DMAPP). IPP and DMAPP are derived from mevalonate (MVA) pathways occurring in the cytoplasm and/or 2-C-methyl-d-erythritol 4-phosphate (MEP) pathways occurring in the plastid. The mevalonate pathway is utilized by photosynthetic microorganisms, including microalgae and plants; the 2-C-methyl-d-erythritol 4-phosphate pathway, on the other hand, is predominantly used by bacteria and fungi. The carotenoid biosynthesis pathway involves the condensation of three IPP molecules and one DMAPP molecule, which produces a C20 geranylgeranyl diphosphate (GGPP) molecule that serves as a precursor to carotenoid biosynthesis. Lycopene is then produced in the next step, which is a common intermediate for the biosynthesis of almost all downstream C40 carotenoids [31]. Figure 2 shows the general biosynthetic pathways of microbial carotenoids.

Various microorganisms can produce carotenoids from natural sources, including bacteria such as *Escherichia coli*, *Dietzia natronolimnaea*, *P. carotinifaciens* or *Pseudomonas putida*; yeasts such as *X. dendrorhous*, *Yarrowia lipolytica*, *Saccharomyces cerevisiae*, *Pichia pastoris*, *Sporiodiobolus pararoseus* and *Rhodotorula mucilaginosa*, filamentous; fungi such as B. trispora; and algae such as *Chlorella zofingiensis*, *D. salina* and *Coelastrella striolata* [32].

The use of waste for the production of various compounds is the focus of interest of many researchers. In particular, the production of carotenoids is coupled with the production of lipids by solid-state fermentation of waste substrates by means of oleaginous yeasts. These yeasts can accumulate a large percentage of lipids and simultaneously produce carotenoids starting from C5 and C6 sugars [33,34]. The multiplicity of waste results in a heterogeneous nutrient content; therefore, there are different microorganisms capable of producing pigments following the fermentation of waste. Fruit waste can be used as a culture medium to produce Rh-induced β-carotene. Mucilaginous [35].

Certain yeasts, such as *S. roseus* and *S. pararoseus*, have the ability to synthesize both carotenoids and extracellular enzymes, such as lipases and cellulases. These strains have the potential to be used in the biotechnological production of carotenoids using inexpensive substrates such as agro-industrial wastes, including lignocellulose [35]. Recent studies have demonstrated the feasibility of developing a new bioprocess for producing β-carotene from the xylose fraction of lignocellulosic biomass using an engineered strain of *S. cerevisiae* [21,36].

### 2.2. Astaxantine

**Figure 3:**
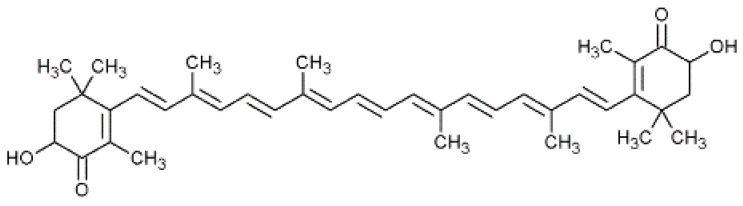
Structural formula of astaxanthin.

Astaxanthine (Figure 3) is a reddish keto-carotenoid belonging to the xanthophyll family. The specific chemical structure with four oxygen atoms makes astaxanthin a xanthophyll with two distinctive characteristics: (1) a peculiar amphipathic character (hydrophobic in the central polyene chain and hydrophilic at the two ends), a property which makes the positioning of the molecule particularly favorable and effective transversely to the phospholipid bilayer of cell membranes; (2) an antioxidant power to possess a significantly higher antioxidant capacity compared to other molecules, including those with similar structures, due to their potent ability to neutralize oxygen radicals (ROS), due to the coordinated action of the conjugated double bonds and ketone groups. Astaxanthin (3,3′-dihydroxy-β-carotene-4,4′-dione) contains two chiral centers, each of which can assume two stereoisomeric conformations for a total of three possible combinations. In nature, when microorganisms grow in a comfortable environment, the production of astaxanthin is hindered. Conversely, astaxanthin accumulation increases when microorganisms grow in uncomfortable environments [37]. The main fungal producer of this compound is the yeast *Xanthophyllomyces dendrorhous* (with Phaffia rhodozyma as its anamorphic state), which mainly synthesizes the (3R, 3′R) isomer [38]. The market for astaxanthin is currently valued at around USD 647 million and is expected to grow at an annual rate of 8.3 to 16.8%, reaching USD 880–968 million by 2026. The main producers of astaxanthin are microalgae such as *H. pluvialis* and *C. zofingiensis* [39,40,41], yeast (*P. rhodozyma* and *Rhodosporidium* spp.) [39,42], easts such as *P. rhodozyma* and *Rhodosporidium* spp., as well as bacteria, including *Paracoccus* spp., *Agrobacterium* spp., *Sphingomonas* spp. and *Pseudomonas* spp. [43]. The heterobasiomycete X. dendrorhous yeast has a productivity of 350 mg/L of astaxanthin in 800 L fermenters; these yields are determined by the ability of the microorganism to accumulate large quantities of astaxanthin. The production of astaxanthin can be implemented following the optimization of the fermentation conditions. The parameters with the greatest influence are the pH, the carbon source, the concentration of nitrogen and from Cu^2+^ and the addition of micronutrients such as vitamins and trace elements [44]. *E. coli* bacteria are not capable of naturally producing carotenoids. However, genetic engineering can be used to introduce carotenogenic genes that enable the production of astaxanthin. Recent studies have shown that *Brevundimonas* sp. can heterologously express β-carotene ketolase (CrtW) and *Pantoea agglomerans* can express β-carotene hydroxylase (CrtZ). Additionally, genetic modification of the chaperones groES and groEL can further enhance astaxanthin production. After 60 h of fermentation in shaken flasks, these modifications have allowed for the production of 1.18 g/L of astaxanthin [25,45,46].

### 2.3. Zaexanthina

**Figure 4:**
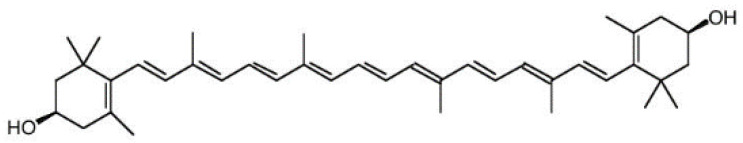
Structural formula of zeaxanthin.

Zeaxanthin (Figure 4) and lutein are two important carotenoids that play a role in protecting the eyes from solar radiation. These pigments are also found in many types of bacteria, and optimization of fermentation conditions has been carried out to obtain higher zeaxanthin titers. *M. aestuarii* KYW614T is a wild-type bacterial strain that can produce the highest concentration of zeaxanthin at 12,000 μg g^−1^ DCW, while *P. zeaxanthinifaciens* ATCC 21588 can produce up to 11.63 mg L^−1^ of zeaxanthin [47,48]. Techniques such as mobilization and continuous fermentation can be used to enhance zeaxanthin production in these bacteria [49].

Through genetic engineering, *E. coli* can also produce zeaxanthin. However, only a few metabolic engineering studies have been conducted on naturally occurring zeaxanthin-producing microorganisms, and few zeaxanthin pathways have been expressed in heterologous hosts. To increase zeaxanthin production via metabolic engineering, it is important to thoroughly investigate native zeaxanthin producers, allowing for the selection and combination of hosts and gene sources to create new zeaxanthin-producing microbial factories [49].

### 2.4. β-Carotene

**Figure 5:**
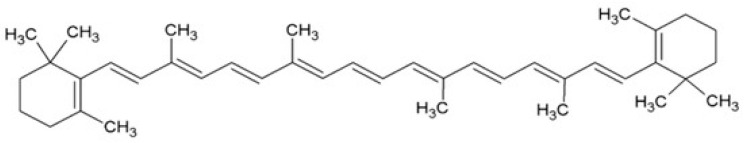
Structural formula of β-carotene.

Beta-carotene is a natural pigment that is commonly found in nature, with the β-isomer being the most stable. When consumed, it can be converted to vitamin A in the human body, playing an important physiological role in tumor prevention, antioxidant, vasoprotective and immunostimulant activities. Despite the fact that β-carotene is naturally produced by fungi, yeasts, some bacterial species, algae and lichens, the production process is expensive. Therefore, using waste materials as a substrate for cultivating β-carotene-producing microorganisms can increase productivity while reducing costs. The chemical formula of β-carotene is C40H56 (as shown in Figure 5), and like other carotenes, it is highly unsaturated and therefore insoluble in water, slightly soluble in ethanol and ether, soluble in benzene and oil chloroform, with a melting point of 176–180 °C [50]. The fungus *B. trispora* is the main source of β-carotene production, with optimization of the culture and growth conditions significantly affecting the yield and biosynthesis of β-carotene from *B. trispora*, with production rates reaching up to 78.0 mg/g DCW [51].

Fungi are the most suitable microbial source for solid-state fermentation due to their lower moisture requirements [52]. Among fungal strains, *Blakeslea trispora* filamentous fungi have been found to produce the highest yield of β-carotene. Lipolytic yeast is another promising source of natural β-carotene [52]. In a study by Akram et al. engineered strains of *Y. lipolytica* produced 797.1 mg/L of β-carotene as an astaxanthin intermediary [52]. The production of β-carotene was successfully accomplished by inducing the expression of crtI and crtYB genes from *X. dendrorhous*, while regulating the expression of other enzymes, such as GGS1/crtE and HMG1, and inhibiting the enzyme SQS1. Furthermore, another research work showed that the combination of genetic manipulation and crop optimization resulted in a yield of 4.5 g/L of β-carotene in a 5-L fermenter, which illustrates the impressive capacity and versatility of *Y. lipolytica* in the biosynthesis of carotenoids [49]. Zhang investigated the ability to produce carotenoids and accumulate fat in six different oily yeasts grown on straw waste. Among these, *R. toruloides* allowed production of 24.58 ± 1.88 mg/L of carotenoids. The lignocellulosic waste must be suitably hydrolyzed in order to be used as a fermentation substrate. Furthermore, based on the nutritional characteristics of the strain used, the fermentation processes must be suitably optimized. Sharma et al. optimized a fermentation process using agro-industrial wastes (such as onion skins, potato skins, mung bean skins and pea pods) as substrate for *R. mucilaginosa* MTCC 1403 [12]. From their study, it emerged that the aeration of the medium affected the production of beta-carotene. Hladnik et al. proposed an integrated process for the extraction, isolation and concentration of β-carotene from *Rhodotorula glutinis*, grown in (processing) effluents [53]. Overall, their data show promising results for a downstream biorefinery cascade process, reducing the damage to the environment and conferring added value to the primary economic activity. Klempovà et al. (2020) have optimized a fermentation process (in solid state on agro-industrial waste (brans, spent malt grains, distiller grains, etc.) with a production of 261.5 mg/Kg of β-carotene with the use of M. wosnessenskii [54]. Contextually with the production of pigment, this microorganism produced a quantity of gamma-linolenic acid of 10.7 g/Kg. Nurkanto et al. studied the ability of *P. rhodozyma* to produce pigments in different substrates (medium containing glucose, soil containing molasses waste, soil containing coconut waste) and found a high sensitivity of the yeast to the production of different pigments in different soils [55]. The highest amount of β- carotene was obtained in a medium containing coconut waste. Fermentation was performed in batches, and pigment determination was performed by HPLC.

At present, the knowledge regarding the utilization of lignocellulosic biomass by anoxygenic photosynthetic bacteria is limited, despite their multiple applications and the nutrient-rich biomass they offer. This restricted comprehension impedes the usage of lignocellulosic waste as a substrate for phototrophic bacteria. In a recent investigation by Patthawaro et al., the growth and products of the photosynthetic bacterium R. faecalis PA2 were studied under light conditions utilizing diverse lignocellulosic waste suspensions [17]. These suspensions, comprising rice straw, bagasse, coconut flour, soybean meal, corncob, palm husk fiber and spent coffee grind, were employed as the sole substrates without additional nutrients and were prepared through filtration or boiling. The selected strain demonstrated the capability to thrive in lignocellulosic waste suspensions without requiring heat pretreatment. Soybean meal exhibited the highest carotenoid productivity of 33.11 mg/L/day among the tested substrates.

### 2.5. Lycopene

**Figure 6:**
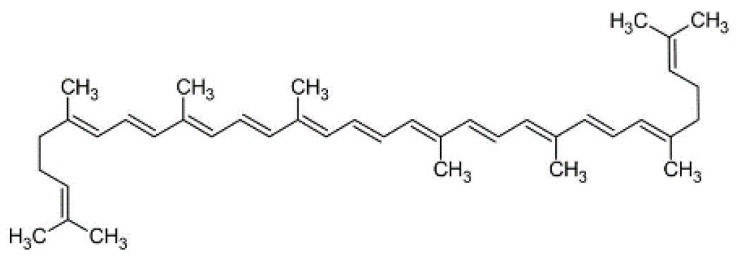
Structural formula of Lycopene.

Lycopene (Figure 6) is a dark red carotenoid found in many ripe red fruits and vegetables belonging to the C40 terpenoids. Unlike other carotenoids, it lacks the terminal β-ionic ring in its structure and does not have provitamin A activity. Lycopene can exist in both cis and trans isomeric forms due to double bonds in its structure, with the transform being the most prevalent in nature [56]. While commercial lycopene is mainly extracted from tomatoes, this method is costly and harmful to the environment. Microbial fermentation is a primary method for lycopene production [57]. There are many reports of lycopene biosynthesis by microorganisms, including *Streptomyces chrestomyceticus*, *Candida utilis* [58], *Blakeslea trispora* [59,60], *Phycomyces blakesleeanus* [61] and a *Flavobacterium* sp. [62] have been described as lycopene-producing microorganisms. *Blakeslea trispora* has received particular attention from researchers. Supercritical carbon dioxide extraction of lycopene from the dried biomass of *B. trispora* mating cultures using acetone as a carrier was efficient with good lycopene extraction efficiency [63]. A biotechnological process for producing lycopene from *B. trispora* mating cultures has been developed, with the addition of vitamin A, acetate, and piperidine during fermentation showing promise for lycopene production [63]. Erkmen and Sevgili et al. conducted a study on the fermentation of Blakeslea trispora to produce lycopene using different substrates [60]. The researchers achieved a maximum lycopene concentration of 944.8 mg L^−1^ through fermentation in bioreactors with 4% glucose and 1.0% sunflower oil. Lycopene production is mainly attributed to the zygospores of *B. trispora*, with the highest intracellular lycopene found in the total dry weight of biomass being correlated with the highest level of zygospores. The presence of substrates containing linoleic acid compounds resulted in high lycopene production. Additionally, lycopene and β-carotene production increased when cultures of Blakeslea trispora were supplemented with n-hexane and n-dodecane as oxygen carriers due to higher dissolved oxygen concentrations. Lycopene biosynthesis generally starts in the first growth phase, even in trace amounts, and the amount of lycopene increases continuously from 2 to 7 days [64]. In Kang et al. research, high-yield production of lycopene was achieved by metabolic engineering of *Deinococcus radiodurans* R1, an extremophile microorganism, using corn liquor (CSL) and glycerol as substrates [65]. The engineered strain was subjected to fed-batch fermentation, which resulted in the production of 722.2 mg/L (203.5 mg/g DCW) of lycopene with a yield and productivity of 20.3 mg/g glycerol and 6.0 mg/L/h, respectively, from 25 g/L of CSL and 35.7 g/L of glycerol. Wang et al. developed a novel fermentation medium for the production of lycopene from *S. cerevisiae* using wastewater residues and biomass supplemented with 3 g/L of yeast extract and D-galactose [66]. According to Wang et al., the use of a novel fermentation medium supplemented with yeast extract and D-galactose led to a 22.4% increase in lycopene production compared to traditional fermentation in shaken flasks (*p* < 0.05) [66]. The researchers tested a continuous autocycle strategy in agitated flasks, and the average lycopene production of the first five cycles showed no significant differences compared to the initial batch. Upon scaling up to a 70 L fermenter, the mean lycopene production reached 5.88 ± 0.15 g/L over three cycles, which was 22.25% more than the initial batch (*p* < 0.05). This study is the first to report the use of a continuous cycle fermentation process for lycopene production. Other microorganisms, including *Escherichia coli*, *Streptomyces avermitilis*, *Saccharomyces cerevisiae* and *Yarrowia lipolytica*, can also be genetically engineered for lycopene production, with the highest reported lycopene content in *S. cerevisiae* being 73.3 mg/g DCW [67].

## 3. Betalains

Betalains, derived from tyrosine, are the primary compounds responsible for the red color displayed by flowers, fruits and other plant tissues. These pigments are mainly found in plant groups of the *Caryophyllales order*, such as *Amaranthaceae* (Beta vulgaris), *Cactaceae* (*Opuntia*, *Pitaya*, or *Pitahaya*), *Nyctaginaceae* (*Bougainvillea*), *Phytolaccaceae* (*Phytolacca americana*) and *Portulacaceae* (*Portulaca grandiflora*) [68,69,70].

**Figure 7:**
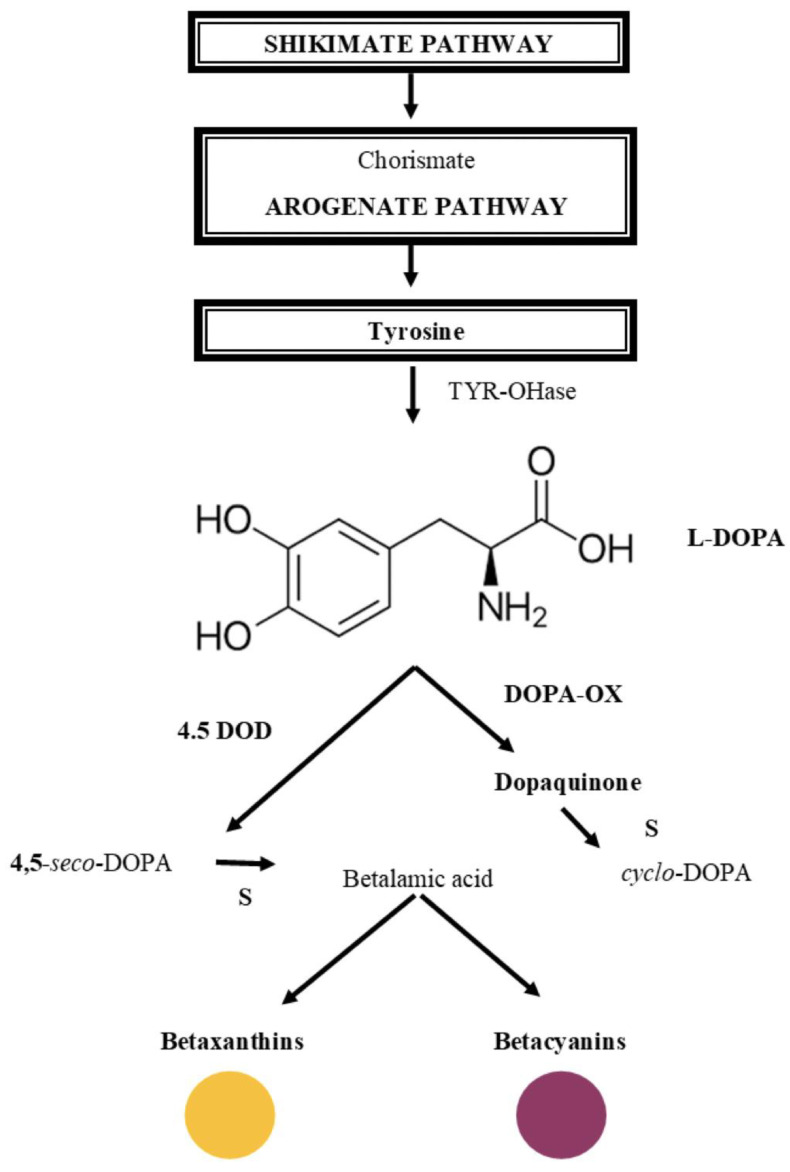
Schematic representation of selected pathways of betalains biosynthesis.

Betalains accumulate in vacuoles of plant cells, predominantly in the edible parts of plant tissues, such as the epidermal and subepidermal layers, along with other phytochemical compounds [71,72,73]. In the UV-visible region, betaxanthin and betacyanin are detectable at the maximum absorption wavelengths of 480 and 540 nm, respectively [70]. These pigments are classified into two structural subgroups based on the ligands they contain: beta-cyanins (red-violet pigments) and betaxanthines (yellow-orange pigments). The chromophore, betalamic acid, is common to all betalain pigments, and the residue added to it determines its classification as betacyanin or betaxanthin. These pigments are immonium conjugates of betalamic acid with cyclo-dihydroxyphenylalanine (cDOPA) glucoside and amino acids or amines, respectively (Figure 7) [74]. The red color of flowers, fruits, and other plant tissues is primarily due to the presence of betalains. Betalains, which are derived from tyrosine, are predominantly found in the order Caryophyllales and in plant families such as *Amaranthaceae*, *Cactaceae*, *Nyctaginaceae*, *Phytolaccaceae* and *Portulacaceae*. Betalain compounds accumulate in plant cell vacuoles, particularly in edible parts of plant tissues, accompanied by other phytochemical compounds. Betacyanins and betaxanthins, which are distinguishable by their maximum wavelength absorption in the UV-visible region, are the two main subgroups of betalains. Betalamic acid is the chromophore common to all betalain pigments, and their classification into betacyanin or betaxanthin depends on the residue added to it. Although betalains can be produced by genetically engineered microorganisms, their practical use is limited by their instability to various factors such as pH, oxygen, water activity, light, and metals. Nonetheless, fermentation studies have shown that betalain can be produced from waste used as a culture medium, with S. cerevisiae being the preferred microorganism for betalain production. The first complete microbial production of betanin in *S. cerevisiae* was demonstrated by Grewal et al. using glucose, with a yield of 17 mg/L from 10 g/L of beet extract [75].

Betalains are naturally occurring pigments found in various plant tissues, and their use is limited due to their instability in response to different factors [76]. Genetic engineering of microorganisms has enabled the production of betalains as pigments, and studies have shown that betalains can be produced by fermentation using waste as a culture medium. Saccharomyces cerevisiae has been extensively studied and engineered for betalain production, and Escherichia coli has also been modified for de novo biosynthesis of betalamic acid, resulting in the production of various betaxanthins. A coculture system of these two microorganisms has also been established for the production of histidine-betaxanthin. Higher plant sources of betalains have also been explored, including beet cell cultures (*Escherichia coli* and *Saccharomyces cerevisiae*) [59,60], and higher plants indicate new potential sources of these pigments. However, their productivity is much lower than that of beetroot, which can produce up to 0.5 g of betanin per kg of roots [77,78].

## 4. Violacein

**Figure 8:**
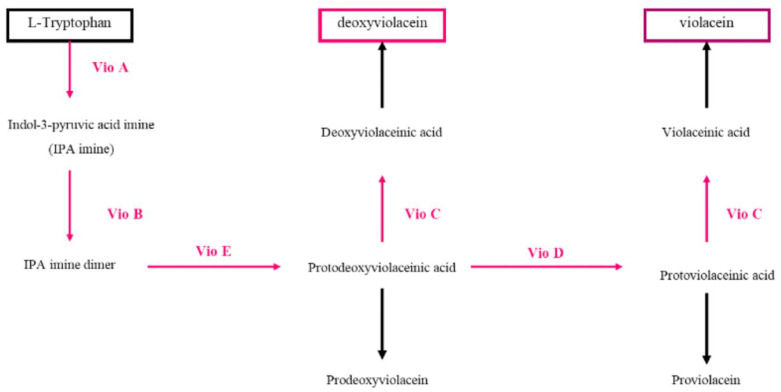
Schematic representation of violacein biosynthetic pathway.

Figure 8 depicts the biosynthetic pathway of violacein that involves five enzymes: VioA, VioB, VioC, VioD and VioE [79]. The pathway starts with the conversion of L-tryptophan to indole 3-pyruvic acid (IPA) imine by VioA, a tryptophan-2 flavin-dependent monooxygenase. The IPA imine is dimerized into a transient imine dimer by VioB and then converted to protoviolacenic acid (PDVA) by VioE. PDVA is further converted to protoviolacenic acid (PVA) by VioD, an NADP-dependent oxygenase. Finally, VioC, another NADP-dependent oxygenase, adds a hydroxyl group on the C2 position of the other indole ring, resulting in the formation of violacenic acid (VA). Spontaneous oxidative decarboxylation of VA leads to the production of the final product, violacein. When the entire vioABCDE operon is expressed, a mixture of violacein and deoxyviolacein, known as “crude violacein”, is obtained [79,80]. Several phylogenetically diverse bacteria found in various environments, including oceans, glaciers, rivers and soil, produce this substance as a secondary metabolite. It possesses numerous biological activities, including potent inhibition of Gram-positive pathogens [81]. Due to its essential antiparasitic, antimicrobial, and antitumoral properties, it is deemed a significant aromatic compound [82,83]. Several bacteria, including *Chromobacterium violaceum* [84,85,86], *Duganella* sp. [87,88], *Pseudoalteromonas luteoviolacea* sp. [89,90] and *Massilia* sp. [91], produce violacein. Violacein exhibits multiple biological activities, such as strong inhibition of Gram-positive pathogens, and is considered an important aromatic compound with essential characteristics. Recent studies have explored the use of agro-industrial waste as a substrate to produce violacein from microorganisms. One study used soybean meal as a cost-effective growth medium for the production of violacein by *Chromobacterium violaceum*, demonstrating the potential of soybean meal as a substrate for economic and sustainable growth medium [92]. Violacein is a pigment synthesized by Gram-negative bacteria, mainly *Chromobacterium violaceum* but several microorganisms are known, including: *Duganella* sp., *Pseudoalteromonas* sp., *Iodobacter* sp. and *Massilia* sp., produce violacein [93]. Several strains of psychrophilic bacteria belonging to the genus Rugamonas were also found to produce violacein [86]. Moreover, violacein production has been demonstrated in several other bacteria, such as *Pseudoalteromonas* sp. 520P1, *V. natriegens*, *C. glutamicum*, *E. coli*, *Y. lipolytica* and *D. violaceinigra*.

###  Oxiviolacein and Deoxiviolacein

Various microorganisms, including *Chromobacterium violaceum*, *Janthinobacterium lividum*, *Duganella* sp. and *Pseudoalteromonas* sp., produce the purple pigment violacein and its derivative, deoxyviolacein [94]. However, deoxyviolacein is produced in insignificant amounts compared to violacein in *Janthinobacterium lividum* and *Duganella* sp. B2 [95]. The VioD protein can be removed from the violacein pathway to generate deoxiviolacein without a hydroxyl group. Studies have shown that deoxiviolacein exhibits better photostability than violacein but can be toxic to HepG2 cell lines, and its impact is dose-dependent [95]. Another derivative, oxyviolacein, has been produced from the derivative of tryptophan, containing one more hydroxyl group than deoxiviolacein [79]. This extra hydroxyl group has elevated its efficacy against human pathogens such as *Staphylococcus aureus* [95]. The pigment violacein is a blue-purple hue made up of two tryptophan molecules that condense to form a bisindole. Various bacteria residing in different environments, such as oceans, glaciers, rivers and soil, synthesize this secondary metabolite. It has been found to possess several biological activities, including potent inhibition of Gram-positive pathogens [81]. Violacein is considered significant due to its antiparasitic, antimicrobial, and antitumoral characteristics [82,83]. *Chromobacterium violaceum* is the first and most extensively studied bacterium known to produce violacein [84,87,88], though other microorganisms like *Pseudoalteromonas luteoviolacea* sp. [89,90] and *Massilia* sp. [91]. *Massilia* sp. also synthesizes this pigment. A recent research project effectively utilized soybean meal, a readily accessible and highly nutritious agricultural waste, as a growth medium for the economic and environmentally friendly production of violacein by *Chromobacterium violaceum*. The research highlighted the potential of soybean meal as a cost-effective growth medium for violacein, as improving the fermentation conditions resulted in greater production [92].

## 5. Prodigiosin

**Figure 9:**
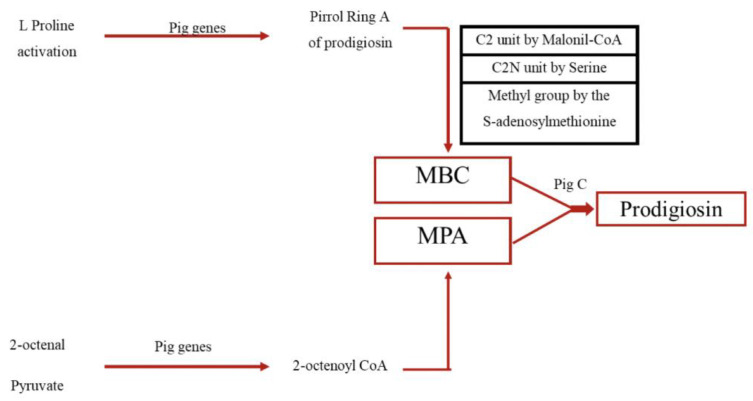
Biosynthetic pathway of prodigiosin.

The red pigment Prodigiosin (PG) is mainly derived from secondary metabolites of microorganisms, particularly Serratia marcescens, and is a tripyrrole molecule consisting of pyrrole (ring A), 3-methoxypyrrole (ring B) and 2-methyl-3-pentylpyrrole (ring C), with the chemical formula C20H25N3O. Although it can also be chemically synthesized, the low efficiency of this process makes microbial fermentation the preferred method for large-scale production for practical applications. Prodigiosin has potential uses in medicine, such as anti-cancer and antimicrobial properties, as well as in electronics, due to its electrical conductivity. The biosynthesis of prodigiosin involves the formation of two critical intermediates, 2-methyl-3-n-amylpyrrole (MAP) and 4-methoxy-2,2′-bipyrrole-5-carbaldehyde (MBC), via a bifurcated pathway, with MAP formed in a three-step reaction from the initial precursor, 2-octenoyl CoA, and MBC synthesized from l-proline (Figure 9). *Serratia marcescens* is the main producer of PG through microbial fermentation. However, there are multiple pathways for the synthesis of the intermediates MAP and MBC, and they involve the activation and conversion of various compounds such as l-proline, malonyl CoA, serine and S-adenosylmethionine. It is also noteworthy that prodigiosin production is not limited to S. marcescens, but has also been observed in other bacteria such as *S. nematodiphila*, *S. plymuthica*, *S. rubidaea*, *Pseudoalteromonas rubra*, *Vibrio* sp., *Janthinobacterium*, *Pseudomonas putida*, *Streptomyces coelicolor* and *Hahella chejuensis* [96,97]. This suggests that the biosynthesis of prodigiosin may be a conserved mechanism among various bacterial species.

The high cost of production and the complicated process of purification and separation are major challenges that limit the practical application of prodigiosin [98]. Therefore, there is a need to increase production efficiency and reduce production costs. One potential method that has been investigated involves the utilization of marine chitinous waste (MCW) as a carbon/nitrogen source for the production of prodigiosin through bacterial fermentation using *Serratia marcescens* strains [99]. Specifically, demineralized shrimp shell powders (de-SSP) have been identified as viable MCW sources for this purpose. Through a 15 L bioreactor system, the maximum yield of prodigiosin (6200 mg/L) was achieved during fermentation by utilizing 5 L of a culture broth containing 1.60% C/N sources, 0.02% K2SO4 and 0.05% K2HPO4, while maintaining an initial pH between 6–7.

## 6. Melanin

**Figure 10:**
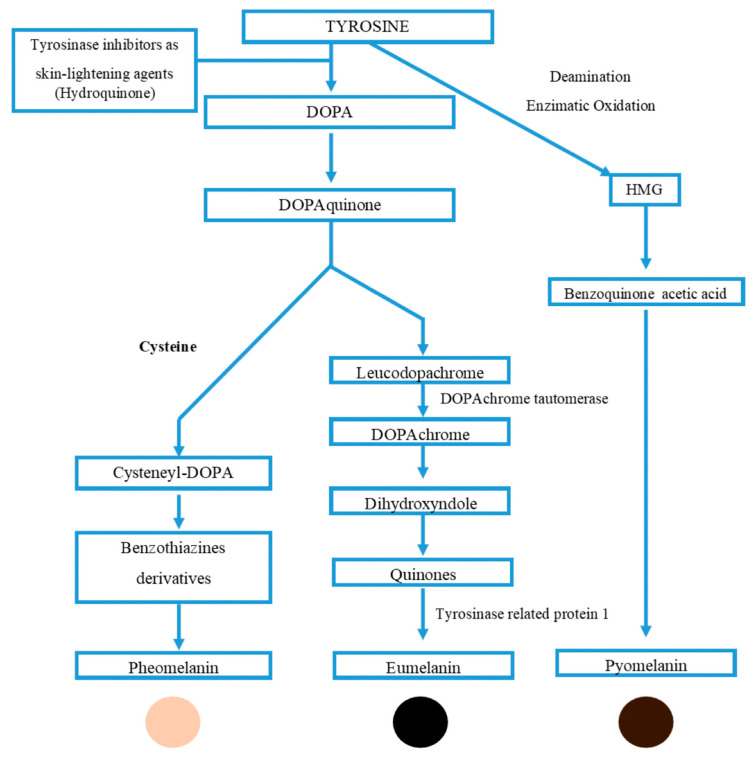
Schematic representation of melanin biosynthetic pathway.

Melanin usually emerges dark brown or black; the pigment derives its name from “melanos”—an ancient Greek word for black [100]. Melanin is a pigment that is widely distributed in nature and plays important roles in a variety of organisms. In animals, including humans, eumelanin is the most common type of melanin and is responsible for the black-to-brown coloration of skin, hair and eyes. It is derived from the oxidative polymerization of tyrosine derivatives such as l-Dopa. In contrast to eumelanin, pheomelanin is primarily present in red hair, freckles or feathers, and it is distinguished by the presence of sulfur in its chemical structure. Pheomelanin is derived from the precursor molecule, 5-cysteinyl-Dopa. The definition of melanin is commonly described as “a polymer composed of phenolic or indolic compounds that undergoes oxidation and subsequent polymerization of intermediate phenols and their resulting quinones” [101]. Allomelanin is the type of melanin found in plants, fungi =and bacteria, which shares a similar chemical composition and formation pathway to eumelanin in animals. Various microorganisms are capable of producing different types of melanin, including eumelanin, through a pathway similar to the mammalian melanin pathway (shown in Figure 10).

Overall, the use of microorganisms to produce melanin has several advantages over traditional methods using plants or animals, as well as over synthetic methods. Microbial production is not affected by seasonal fluctuations, can adapt to different growth conditions, and can be optimized using various fermentation parameters to achieve high yields. In addition, using renewable sources such as fruit waste, carrot peel extract, or marine waste, as demonstrated in the studies mentioned, can also make the process more sustainable and environmentally friendly.

Synthetic melanin polymers can present environmental and quality concerns, as with other pigments. In comparison, microbial-derived melanin offers many advantages over synthetic and animal- or plant-based melanin production methods. The growth and mechanisms of microbes used for melanin production can be modified according to the soil composition and growth conditions provided, allowing for independent production from seasonal fluctuations that may interfere with other bio-based methods, such as marine and vegetable melanin production [102].

Tarangini and Mishra optimized the production conditions of melanin by *Bacillus saphensis* using a cheap fruit waste extract [103]. Under optimal conditions (pH 6.84 and temp 30.7 °C), the authors reported a significant yield of approximately 6.96 mg/mL. Müjdeci (2022) used response surface methodology to optimize the fermentation parameters for melanin production by *Aureobasidium pullulans* NBRC 100,716 using carrot peel extract [104]. The estimated concentrations of intracellular, extracellular, and total melanin under optimal conditions were 2.44  ±  0.05 g/L, 1.95  ±  0.47 g/L, and 4.22  ±  0.74 g/L, respectively. Lin et al. (2022) used Aureobasidium pullulans strain HIT-LCY3T to produce pullulan and melanin from industrial potato starch waste. Under optimal conditions, the pullulan and melanin yields were 23.47 g/L and 18.98 g/L, respectively [105]. Restaino et al. (2022) conducted a study to enhance extracellular melanin production by *Streptomyces roseochromogenes* ATCC 13,400 using *Posidonia oceanica egagropili* as a renewable source [106]. The researchers added different amounts of the egagropili powder to a culture medium containing glucose, malt extract, and yeast extract to assess its effect on melanin biosynthesis. Results indicated that the addition of 2.5 g L^−1^ of egagropili powder during 120 h of growth at 26 °C, pH 6.0, and 250 rpm in a stirred flask increased melanin production up to 3.94 ± 0.12 g L^−1^. In 2-L batches, they obtained a concentration of 9.20 ± 0.12 g·L^−1^ in 96 h, with productivity of 0.098 g·L^−1^·h^−1^. Further studies showed that the lignin–carbohydrate complex and holocellulosic components of egagropyls worked synergistically to enhance melanin production.

## 7. Application of Pigments

**Figure 11:**
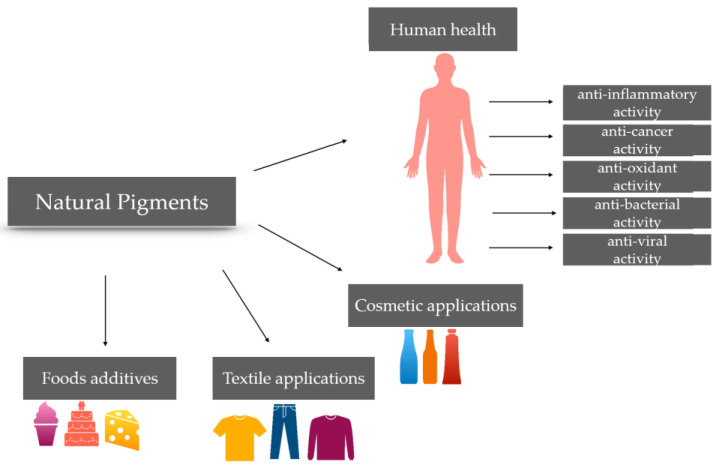
Summary diagram of the applications of natural pigments.

In the food industry, natural pigments have become increasingly popular in various fields, including pharmacology, toxicology, textile and printing industry, as well as the dairy and fish industry. Currently, it is a frequent occurrence to observe all principal categories of natural pigments being employed in at least one area of the food sector (Figure 11).

### 7.1. Food Industry

The food industry encounters obstacles such as expenses, usage, procedures and excellence when using natural pigments. Despite microbial pigments providing a better option compared to botanical pigments for synthetic colors, they may cause disagreeable flavors and aromas and have weaker coloring power. Moreover, substituting synthetic colors with natural colors is demanding as only a limited number of natural colors are permissible for use in food. Natural colors are also vulnerable to environmental factors such as light, pH, temperature, UV, oxygen and heat, leading to color fading and reduced product longevity [10]. In the last few years, many studies have been focused on natural food pigments from microorganisms.

The use of natural pigments from microorganisms has been a popular area of research due to the advantages of scalability, non-seasonality, and higher yield per hectare. However, natural colors are generally more costly to produce than synthetic colors, and a greater quantity of raw materials is necessary to generate the same quantity of natural colors as synthetic colors. Additionally, higher dosages of natural colors are often required to achieve the desired hue, further driving up costs [10].

Despite these challenges, natural colors remain a popular area of research due to their potential benefits. The ratio is 5:1 in terms of costs; in confectionary items, this ratio could increase up to 20:1 [107].

To create the same amount of natural colors as synthetic colors, a significant quantity of raw materials is necessary. This implies that achieving the desired hue with natural colors typically requires higher doses, resulting in increased expenses [10]. In order to make microbial pigments more affordable, it is important to explore and develop more cost-effective techniques for their recovery and separation. So far, the most effective method for extracting these pigments has been through the use of non-ionic resins. Wang et al. have demonstrated the efficacy of this technique in the separation and purification of prodigiosin by directly using non-ionic resins from the culture broth. This method eliminates the need for cell separation, resulting in a concentrated and semi-purified product [108].

Natural pigments have potential as food additives, with red being the most commonly used color to attract consumers. Beta-carotene is becoming increasingly important in the food industry as a colorant, with concentrations ranging from 2 to 50 ppm in juices, drinks, butter, margarine and cheese [109]. The goal is to meet the demand of the global food market by extracting β-carotene from microbial feedstocks instead of using synthetic derivatives. However, there are production issues with this pigment, such as solubility, stability, melting point and low bioavailability, that need to be optimized. Delivering carotenoids by using polymeric nanocapsules may help in removing such obstacles [110]. The yellow zeaxanthin, extracted from *Flavobacterium* spp., has demonstrated antioxidant activity while acting as a colorant in food [111,112]. The same properties were noticed in the orange/deep pink Canthaxanthin pigment from *Bradyrhizobium* spp. [113]. *Aphanizomenon* spp., which belongs to cyanobacteria, is able to produce a blue pigment named phycocyanin, which is used widely in the food and beverage industry [114]. Another well-studied cyanobacterium is Spirulina spp., which has a high phycocyanin content, up to the 20% of its dry weight. The main limitation of phycocyanin is its low resistance to high temperature, as it precipitates; as a result, it obtains the fading of the blue color. This weakness restricts its use in food. The researchers tried to stabilize the proteins overcoming this issue by adding sugars and polyhydric alcohols, which are totally safe for health. Su et al. introduced a prime medium composition for culturing *Serratia marcescens* in order to optimize the production of prodigiosin [115]. Sucrose and glycine were used as carbohydrates for energetic purposes. This escamotage ameliorated prodigiosin’ production. Another boost was added by inorganic supplementation with KH_2_PO_4_; as a result, there was augmented cell growth. The aforementioned steps were essential for obtaining a cheaper production process and reaching an efficient fabrication procedure.

Another step towards the replacement of artificial dies in the food industry is given by the red pigment (PG) generated by *S. marcescens*. However, it has some limitations, likewise solubility and short stability once exposed to diverse pH, light and high temperatures. In order to avoid this problem, the researchers suggested a different delivery system. PG was encapsulated with Kappa-carrageenan and maltodextrin and packaged in a spray-dried formulation to be used for dyeing yogurt, milk and carbonated beverages. The product was enhanced for food utilization [116].

Various Gram-negative bacteria, including *Chromobacterium violaceum* [117], *Janthionobacterium lividum* [118], *Alteromonasn luteoviolacea*, *Pseudoalteromonas luteoviolacea* and *Duganella* spp., have been found to produce violacein, a violet pigment that acts as a secondary metabolite. An interesting study demonstrated that violacein produced by *C. violaceum* could be used as a potential antioxidant stimulating mucosal defense mechanisms [119]. In fact, it was shown that a regular intake of violacein influenced the microbial composition of the gut of rats.

Riboflavin, or vitamin B2, is accepted in dairy products, drinks and baby foods. Riboflavin is especially used for the photosensitized reactions in dairy products and beer. The photosensitivity reactions of riboflavin are also used for meats during chill storage [120]. Moreover, riboflavin is produced using lactic acid bacteria (LAB) for obtaining probiotic foods. *Lactobacillus fermentum* isolated from sourdough was able to produce riboflavin. In these experiments, Lactobacillus fermentum was exposed to the selective pressure of roseoflavin, and the muthans obtained have been analyzed for their ability to overproduce riboflavin. A study conducted on small-scale bread production revealed that incorporating a combination of co-inoculum yeast and *L. fermentum* resulted in an approximately two-fold rise in the final vitamin B2 content [121].

The fermentation of the genus of *Monascus* sp was used to produce rise, food colorants, and fermentation starters for more than 1000 years in China [122]. In addition, it could be added to yogurt fruit to enhance the color of the product. Monascus sp fermented rice was used for flavored milk [123]. Monascus pigments were also used as food colorants. The colorant and its derivative, Monascarubromine, is used in Asiatic meals like red rice and red bean curd as well as in seafood, meat and ketchup [124].

Fungal cultures provide pigments such as carotenoids that are utilized in poultry and fish feeds and are also employed to add color to the skin, egg yolks of poultry, fish flesh and crustacean shells [125]. Industrial production of β-carotene and astaxanthin from microbes is widespread in the food and feed industries. Nonetheless, carotenoids can also be derived from by-products generated by fruit and vegetable processing operations, such as paprika waste [126], tomato peel [127], carrot peel [128] and their residues. Furthermore, anthocyanin pigments obtained from by-products of food processing industries, such as juice or wine, are employed as natural dyes in various foods. For example, blackberry residues [129] and apple peel [130] are substantial sources of natural colorants due to their high anthocyanin content.

### 7.2. Pharmacological and Nutraceutical Industry

The various pigments possess biological functions that can safeguard human health, including but not limited to anti-viral, anti-microbial, anti-cancer, anti-oxidant and anti-inflammatory properties that utilize cytokine pathways and subsequently, immuno-modulating activities through free radical scavenging signaling. Moreover, microbial pigments have significant clinical applications in the diagnosis of diseases such as cancer, leukemia and diabetes mellitus [131]. One of the most significant advantages of microbial pigments is their ability to target cancer cells selectively while leaving healthy cells unharmed. This is due to the specific interactions between the pigment molecules and the biochemical pathways that are active in cancer cells. This specificity makes microbial pigments promising candidates for cancer diagnosis and treatment.

For instance, some microbial pigments exhibit fluorescent properties that can detect cancer cells during surgery or in tissue samples. They can also be used to identify the edges of tumors, allowing surgeons to ensure that all cancer cells are removed.

Furthermore, the antioxidant and anti-inflammatory properties of microbial pigments can aid in lowering the risk of cancer development by reducing oxidative stress and inflammation. They can also potentially have a direct effect on cancer cells, such as initiating programmed cell death [132].

It is established that the production of pigmented secondary metabolites controls the growth of other competing bacteria, constituting an antimicrobial activity. For example, pyocyanin (blue-green pigment) is capable of inhibiting the growth of *Escherichia coli* [133], *P. aeruginosa*, *S. aureus*, *Staphylococcus saprophyticus* and *Enterococcus faecalis* [134].

The bioactive derivatives from bacterial isolates, such as prodigiosin (red), violacein (violet), flexirubin (yellowish-orange), carotenoids (yellow-orange) and pyocyanin (blue-green), have been found to possess various activities, including antimicrobial, antiviral, antitumor, antiprotozoal, antioxidant and anticancer activity [134].

Recent studies have also indicated that these pigments with antimicrobial properties can hinder the formation of pathogenic biofilms [135]. For example, violacein has demonstrated efficacy against methicillin-resistant *S. aureus* (MRSA) strains [136], and it has been shown to prevent the formation of biofilms by Staphylococcus epidermidis. This opportunistic pathogen can form adhesive communities on catheters, which leads to persistent infections and sepsis in hospitalized patients [137].

For these reasons, the combination of antibiotics and anti-microbial pigments could represent a new approach to counteract the propagation of dangerous bacteria.

Overall, red pigments have the highest antimicrobial capacity [138]; astaxanthin and prodigiosin [139] have numerous anti-bacterial and anti-inflammatory applications and are widely used in the pharmaceutical and animal feed industries.

Since, in recent years, new viruses have affected humans, it could be important to investigate new natural compounds with anti-viral action. Violacein showed weak inhibition of the viral replication of Herpes Simplex (HSV), indicating that it provides inhibition of viral duplication, especially for Poliovirus type 2 and Simian rotavirus SA11 [140]. Another study demonstrated the anti-viral effects of prodigiosin, produced by *S. marcescens*, against HSV infection. Data demonstrated that the infection of HSV-1 could determine an increase in NF-kB, but also that the level of TNF-alpha and of NF-kB was downregulated after prodigiosin treatment [141].

The yellow pigments, which belong to the carotenoid family, presented antifungal activity versus fungal pathogens, such as Rhizoctonia solani and Sclerotium rolfsi [142].

Moreover, violacein could be effective against human and plant pathogenic fungi such as *Penicillium expansum*, *Candida albicans*, *Trichophyton rubrum* and *Fusarium oxysporum* [143]. In addition, at high concentrations, violacein has an antileishmanial and antimalarial potential [144].

Natural bacterial pigments have been demonstrated to have potential as chemotherapies, having antitumor functions. Several studies reported the cytotoxic effect of prodigiosin produced by *Pseudoalteromonas* sp against leukemia cells, B-cells and lymphocytes of leukemia patients [145,146]. At the same time, prodigiosin produced from *Serratia marcescens* induced apoptosis in human cancer cell lines [147]. Nunez Selles and colleagues have conducted a study that suggests that mangiferin, a xanthonoid-rich compound extracted from mango trees, may have promising therapeutic effects for various types of cancer such as lung, brain, cervix, prostate, breast cancers and blood malignancies, especially when combined with other anti-cancer drugs [148].

The utilization of natural compounds to combat cancer has shown promise in recent studies. For instance, betalains extracted from beetroot have been shown to possess significant anti-proliferative properties in human cell lines such as MCF7 and MRC-5, making it an excellent candidate for chemotherapy [149]. Similarly, carotenoids from Kocuria sp have demonstrated anticancer activity against MCF-7 breast cancer cell lines [150]. Numerous cell lines have demonstrated the anticancer efficacy of violacein. In a study conducted by Melo et al. in 2000, it was found that violacein exhibited high cytotoxicity to V79 fibroblasts [151]. Overall, violacein is the pigment that showed the greatest activity against cancer and other diseases due to its ability to induce apoptosis through the upregulation of TNF-α expression and the p53-dependent mitochondrial pathway and interfere with the cell cycle [152]. According to reference [153], the strain of *P. aeruginosa* can produce a potent compound called pyocyanin, which has been found to inhibit the growth and promote apoptosis of pelvic rhabdomyosarcoma cells. In addition, the melanin pigment derived from *Streptomyces glaucescens* has been reported to exhibit cytotoxic activity against skin cancer [154].

Antioxidants play a critical role in numerous degenerative pathologies inhibiting and eliminating free radicals and increasing the production of antioxidant enzymes. Several synthetic antioxidants are meant to block the oxidation process, which is potentially detrimental to health. Authors are focusing on discovering natural alternatives with antioxidant capacities [155].

Several studies have reported the protective effects of natural compounds against oxidative stress damage in cells. For example, carotenoids extracted from *Kocuria marina* [150], violacein extracted from *C. violaceum* [156] and pyomelanin from *Burkholderia cenocepacia* [157] have all demonstrated the ability to protect cells through various defense mechanisms. Additionally, two authors have reported that β-carotene can suppress the harmful effects of free radicals in humans [158].

In the last few years, many natural substances, including pigments, have attracted the attention of researchers for therapeutic strategies since they demonstrated their proprieties as anti-inflammatory or immune-modulating agents.

The ankaflavin possessed anti-allergic activity on lung cells [159]. Polyphenolic compounds and anthocyanins obtained by raspberry pomace have been studied for their potential anti-inflammatory capacities [160]. This study showed that anthocyanins, enzymatically extracted from raspberry, were capable of inhibiting lipoxygenase and cyclooxygenase 2 activities, displaying anti-inflammatory properties. Srilekha et al. isolated *Micrococcus* sp, which is a water-pigmented bacteria. It was demonstrated to have a strong anti-inflammatory potential working also as wound healing agent. These characteristics are mainly due to the antibacterial property of the pigment [146].

Egeland et al. proposed a carotenoid as a possible new anti-inflammatory: the fucoxanthin extracted from cyanobacteria. It has anti-cancer and anti-inflammatory properties. Therefore, this bioactive pigment also has proven wound-healing abilities [161]. A phytochemical, limonin (a triterpenoid), extracted from citrus waste, could play an important role as an anti-inflammatory, regulating STAT3/miR-124 signaling pathway and also operating as an antioxidant, anti-cancer and anti-bacterial agent [162]. Kaempferol is a natural flavonoid considered a precious functional food ingredient for therapeutic applications that showed anti-carcinogenic and anti-inflammatory effects [163]. Huynh et al. developed cauliflower by-products, using filamentous fungi undergoing solid-state fermentation to produce prominent amounts of phenolic compounds that increased the extractability of kaempferol glucosidase [164].

Moreover, a study by Lee et al. revealed that rose petal extract obtained from Rosa gallica contains high levels of anthocyanins, polyphenols and flavonoids. This extract was found to exhibit skin anti-inflammatory activity by inhibiting the MAPK signaling pathway and reducing the expression of COX-2 as well as several cytokines [165].

These results show the close correlation between natural pigments and their anti-inflammatory effect by playing a role in the regulation of proinflammatory cytokines.

### 7.3. Cosmetic Industry

Cosmetic products are a blend of biological and chemical compounds. Various microbial compounds are used in the cosmetic industry. Ingredients from biological sources like plants and other organisms constitute a main source of promising products. Bacteria and fungi metabolites represent one of the cheapest, most renewable and novel sources of natural goods. Microbes have a lot of potential, and only of few of them are industrially employed for cosmetics [166]. However, the major advantage of using microbial pigments is their biocompatibility with high-quality products and a low environmental impact.

Microbial pigments are applied for different tints in cosmetics, especially for skincare products. For example, natural melanin, probably the most used pigment in the cosmetology sector, extracted from *Streptomyces bellus*, is used for the production of a bio-lip [167]. Melanin pigments are also used as a significant component of sunscreens and beauty care products with sunscreen since this pigment may disperse about 99% of absorbed UV light [154]. Melanin pigment gives color to the skin and has an important function in protecting the skin from the harmful effects of UV light, protecting against carcinogenesis. Nevertheless, excessive production of melanocytes could cause hyper-pigmentation and premature aging. Astaxanthin, a carotenoid obtained from *H. pluvialis*, reduces melanin by 40% in epidermal cells, helping to defend the skin from premature aging. For this reason, astaxanthin is a new fundamental component of after-sun lotions [168]. Instead, astaxanthin extracted from *Haematococcus pluvialis* regenerates skin tone and is used in many anti-age creams [169]. Moreover, the bio pigments produced by *Staphylococcus xylosus*, indicated an activity against the sun’s rays, so it could be used as an ingredient in cosmeceuticals products [170]. The red pigment prodigion has been shown to increase the protective factors of sunscreens by around 20–65%, and it is also used for augmenting the anti-inflammatory activity and protective factors of aloe vera creams [96]. The carotenoids family, including astaxanthin, lycopene, β-carotene and canthaxanthin, are being marketed in combination with cosmetics due to their antioxidant additive properties [171]. Moreover, violacein is an important element for cosmetic products such as antiperspirants, lipsticks, eye makeup and all products that have to stay in contact with human skin for a long time due to their ability not to cause injury or damage to epidermis cells [117].

### 7.4. Textile Industry

The practice of utilizing synthetic dyes for coloring clothing garments has been in use for several centuries. Although these dyes are widely available and produce a vast range of colors at an affordable price, they can release harmful and toxic substances that may cause allergic reactions and other health issues in the human body. Therefore, natural pigments are increasingly being used in the textile industry as they are non-dangerous, non-carcinogenic and eco-sustainable [8].

The red pigment PG obtained from *Vibrio* spp. and *Serratia marcescens* is used to dye fibers such as silk, cotton, nylon and acrylics, even resisting washing and variation of external conditions [172]. Prodigiosin, extracted from *Serratia marcescens*, has been widely used as a colorant in the textile industry [85]. On the other hand, violacein, obtained from *C. violaceum*, was used to color viscose, polyester, pure cotton and pure silk. Moreover, Violacein from *Pseudoalteromonas* sp. is used in large quantities in the textile and toys industry for its environmentally friendly derivatives [173].

Indigo has been a popular textile colorant for blue denim for many years and was initially derived from plant material. However, it is now primarily industrially manufactured from fossil feedstocks. Authors have recently presented a summary of several microbial enzymes that can produce indigo. The article highlights the pros and cons of each biocatalytic method. Despite these efforts, large-scale industrial production of indigo remains unavailable [174].

Additionally, the pigment extracted from *Sclerotinia* sp. has been widely utilized for dyeing pure cotton [175].

### 7.5. Future Perspectives and Conclusive Remarks

Bacteria represent a natural source of diverse pigments. Due to the ability to control their growth, the use of microorganisms to produce dye could constitute a cheap alternative to synthetic pigments. Bacteria can be easily cultivated, and their growth is not seasonal. Moreover, organic waste could be used as culture media. Using microbial pigments as food dye and flavoring agents is not their sole function. They could also be applied as nutraceuticals. In fact, one of the natural pigments’ most interesting applications is the pharmacological one, especially against antibiotic resistance. Studies about their pharmacological application gradually increased during the last few years. Among them, the anti-cholesterolemic, anti-bacterial and anti-inflammatory activities are the most trending in research. Novel applications of natural pigments are focused on pathogenic mechanisms that could be useful for novel approaches against cancer. The microbial production of pigments is beneficial both in economic and environmental terms; moreover, it allows easy control of the cultivation of microorganisms with shorter production times. Synthetic pigments are potentially toxic; therefore, commercial interest in natural pigments has increased. The ability of the microorganism to digest agro-industrial waste reduces the overall cost of production due to the reduction of the disposal cost, the generation of energy by the biogas or biofuel production and the reduction of handling and transport costs. Microbial pigment production faces various challenges, including low yield and safety concerns associated with some pigment-producing opportunistic pathogens. Thus, it is crucial to optimize the production process on an industrial scale to enable the commercial production of microbial pigments.

Although recent data demonstrated that the production of natural pigments is a costless process, it still has some limitations, such as low stability, low yields and a potential danger for toxins produced during the extraction process. For these reasons, the optimization of cultivation conditions and the development of better microbial strains are needed to obtain a more effective purification process. Nevertheless, considering recent developments in the application of agro-industrial waste, low-cost and safe industrial production of natural pigments will be possible in the future. The combination of the colorant action and of the natural antioxidant activity of natural dyes is a winning duet in the food industry. In fact, the pigments would naturally color foods, and they would simultaneously exert a preserving action together with benefits on consumer health.

In this scenario, the cost-effective benefits for the industry will be welcome, but they will be obscured by the benefits for people. Obtaining easily usable, non-toxic, eco-sustainable, cheap and biodegradable pigments represents the future in which researchers should invest.

## Figures and Tables

**Table 1:** Pigments productivity of microorganisms grown in different low-cost media.

Pigments	Microorganism	Waste	References
ß-Carotene	*R. gracilis* ATCC 10788	Potato wastewater	Kot et al., 2020 [11]
Torularhodin, β-carotene, torulene	*R. mucilaginosa* MTCC-1403	Agro-industrial waste	Sharma et al., 2020 [12]
ß-Carotene	*B. trispora* MTCC 884	Fruit and vegetable waste	Kaur et al., 2019 [13]
Total carotenoids	*R. toruloides* ATCC204091	Vegetable waste	Sinha et al., 2021 [14]
Total carotenoids	*R. glutinis* Y1	Vegetable waste	Sinha et al., 2021 [14]
Total carotenoids	*R. rubra* GED8	Whey ultrafltrate	Simova et al., 2004 [15]
Total carotenoids	*R. glutinis*	Salted cheese whey	Kanzy et al., 2015 [16]
Lycopene	*R. faecalis* PA2	Agro-industrial waste	Patthawaro et al., 2020 [17]
Total carotenoids	*R. sphaeroides O.U.001*	Olive mill wastewater	Eroglu et al., 2010 [18]
Torularhodin, β-carotene, torulene	*R. mucilaginosa*	Olive mill wastewater	Ghilardi et al., 2020 [19]
Total carotenoids	*R. toruloides*	Wheat straw hydrolysates	Liu et al., 2019 [20]
Total carotenoids	*L. starkeyi*	Wheat straw hydrolysates	Liu et al., 2019 [20]
ß-Carotene	*S. cerevisiae*	Lignocellulosic biomass	Cheng et al., 2020 [21]
Total carotenoids	*S. roseus*	Spent coffee	Petrik et al., 2014 [22]
Total carotenoids	*P. fermentans*	Rice water	Otero et al., 2019 [23]
Astaxanthin	*H. pluvialis*	Fruit and vegetable waste	Yazgin et al., 2020 [24]
Astaxanthin	*X. dendrorhous*	Food waste	Gervasi et al., 2018 [25]
Prodigiosin	*Enterobacter sp.* PWN1	Casein acid hydrolysate	Poddar et al., 2021 [26]
Melanin	*A. carbonarius*	Fruit and vegetable waste	Arikan et al., 2020 [27]
Violacein	*C. violaceum* UTM5	Pineapple waste	Aruldass et al., 2018 [28]

## Data Availability

Not applicable.
